# Development of mental health indicators at the district level in Madhya Pradesh, India: mixed methods study

**DOI:** 10.1186/s12913-018-3695-4

**Published:** 2018-11-19

**Authors:** Shalini Ahuja, Petra C. Gronholm, Rahul Shidhaye, Mark Jordans, Graham Thornicroft

**Affiliations:** 1 Centre for Implementation Science, H3.08 Health Services and Population Research Department, King’s College London, Institute of Psychiatry, Psychology and Neuroscience, De Crespigny Park, London, SE5 8AF UK; 2 Social Services Research Unit, London School of Economics and Political Science, Houghton Street, London, UK; 3 Public Health Foundation of India, New Delhi, India

**Keywords:** Mental Health, Mental Health Indicators, India, Information Systems

## Abstract

**Background:**

Strengthening routine information systems for mental health can augment scale up of community mental health services in India and other low- and middle-income countries. Currently little routine data is available in Indian settings. This study aimed to develop a core set of indicators for monitoring mental health care in primary health care settings

**Methods:**

By using a sequential exploratory mixed methods design, key mental health indicators measuring service delivery and system performance were developed for the context of Madhya Pradesh, India. The research design involved a situation analysis, and conducting a prioritisation exercise and consultation workshops with key stakeholders.

**Results:**

This study resulted in nine key mental health indicators covering both mental health service delivery indicators and mental health system indicators for Sehore district of Madhya Pradesh. Mean indicator priority scores ranging from 4.48 to 3.78 were reported.

**Conclusions:**

This study demonstrated a phased approach to strengthen routine information systems for mental health at a primary care level in India. We recommend that similar research methods can be applied across comparable settings and these indicators can be adopted as a part of national routine information systems.

## Background

Mental health (MH) indicators summarise data to reflect change in mental health services, their reach, and the populations served. Health information systems are a key building block in strengthening health systems, and indicators are described as key information system tools [1]. Specifically, for mental health and well-being, indicators on suicide, and treatment of substance abuse have been included in the Sustainable Development Goals of Agenda 2030 [2]

In 2015, the United States Agency for International Development, WHO and World Bank met during the Measurement and Accountability for Results in Health summit, and called for action in improving, and hence investing in, health facility and community information systems [2, 3]. Globally there is also a clear need to strengthen routine data collection for mental health cases [4, 5]. These systems are useful at different stages in planning and implementation of mental health care; that is, situational analysis, priority setting, option appraisal, programming, implementation and evaluation [6].

Even though this robust system for routinely collecting MH data is recommended in the WHO Mental Health Action Plan of 2013-2030 [7], few countries have a robust system for routinely collecting mental health data. Lower and middle-income countries (LMICs) in particular face a considerable challenge to strengthen information systems for mental health [4]. Data from the most recent WHO Atlas survey [5] suggested that mental health data are often lacking from most national routine health systems. There has been an ongoing measure to improve quality of information systems globally in the health sector [8]. Routine data tends to be incomplete, inaccurate and are often focused on infectious and communicable diseases or maternal health. Countries are now utilizing information and communication technology to improve quality of information systems for mental health. Measures are taken to also place validation checks to increase culture of information to improve data quality [9].

Mental health data in routine information systems in (LMICs) are considered too unreliable even to calculate essential indicators such as service delivery and system performance. A situational analysis of the status of the health management information systems for mental health in the countries where Emerald project was implemented concluded that countries face considerable challenges within the policy and governance system but also lack capacity in terms of health management information systems (HMIS) experts, infrastructure, supervision support affecting the quality of the mental health data collection, reporting and dissemination. [4].

The current study which was also conducted within the Emerald project from 2014 to 2016, which focusses on strengthening mental health system outcomes in six LMICs including India [10].

In India, for example, the most common method of mental health data collection is through treatment records or case sheets. A recent study in India noted that data on diagnosis in the information systems did not reflect the new ICD-10 system of disease classification [11]. Upadhaya and colleagues also pointed out that in India and other countries mental health data collected through routine monitoring is inadequate and untimely to be of use by policy makers [4].

Therefore, there is an identified need to update, develop and eventually integrate mental health data with the routine health information systems in India [10].

Amongst indicators, the ones measuring coverage have been well documented to evaluate outcomes of mental health programmes in LMICs [12]. Coverage can be studied on a spectrum, ranging from potential coverage, that is whether services are available for patients, to actual coverage, that is whether patients can use services effectively [12].

Previously efforts to strengthen mental health information systems have been made in Ghana, South Africa and Uganda [8, 9, 13], however most of them were generally not sustained. It is believed that lack of evidence base on what to measure and how to measure effectively has hindered scaling and sustaining the initiatives of integrating mental health care with community settings [14].

Consideration of the implementation challenges during the design phase ensures sustainability of the new mental health indicators [9]. Challenges included lack of policies and plans [4], issues with the local capacity and the problems in the workflow mechanisms [15] and insufficient health workforce motivated to collect and more often use the collected data [14].

Countries can determine what to measure by defining their health priorities using priority setting exercises. In research, a priority setting exercise is seen as a social process involving theory, confronting practical obstacles and understanding context to allow decision makers to rate aspects of health services [16]. In the area of health research priority setting has been used, for example, to plan for health care spending in Kenya [16], to reach consensus on prioritising mental disorders in Nepal [17], and to prioritise health conditions to achieve universal health coverage in LMICs [8].

Drawing from these insights, this study aims to develop appropriate and feasible indicators measuring mental health service delivery and system performance through an inclusive process of stakeholder engagement for Sehore district of Madhya Pradesh state, India.

## Methods

### Study design

This study used a sequential exploratory mixed methods research design [18]. A mixed methods research approach draws on both qualitative and quantitative lines of inquiry, to develop and test a new instrument and/or strategy. In a sequential design, one data set builds on the results from the previous data set [19]. A widely used approach within such designs is the exploratory sequential design, where qualitative methodologies are initially used to explore the aspects of the research question, followed by further quantitative examinations guided by the initial qualitative insights.

The use of such an exploratory design in this study was driven by the need to develop mental health service delivery and system performance indicators in an inductive, bottom-up manner, given the current poor acquisition and reporting of pre-existing comparable indicators. Neither of the phases (qualitative or quantitative) was given preference as each step informed the next [20].

Three phases of sequential data collection are described next. In phase 1, a situational analysis tool was used to assess the status of current health management information systems in India. In phase 2, we conducted a prioritisation exercise to select a set of mental health indicators measuring effective coverage and system performance. This was followed by phase 3 in which consultative workshops were used to review whether the proposed indicators were acceptable or if they required further adaptation.

### Settings

The study was conducted at Sehore district of Madhya Pradesh. However additionally various state and national level representatives were consulted during the different phases of this study.

Sehore district is situated in Madhya Pradesh State. Madhya Pradesh is the central state of India and has a population of 72.5 million which is 6% of the total population of India. The state has poor health indicators. Sehore district has a population of 1.3 million and is predominately rural (81%). This district was selected as it is the only district in Madhya Pradesh where the district mental health programme was functional which gave us the platform to develop new mental health indicators. Within the district mental health programme there is one psychologist and one part time psychiatrist catering for the mental health needs of the entire district.

#### Phase 1: Situational Analysis

The situational analysis checklist was developed by three investigators working with the Emerald project [10]. Detailed methodology of the development of the tool has been reported previously [4].

In brief, the situation analysis tool included questions on Health Management Information Systems (HMIS) *inputs* (human resources, availability of mental health indicators), HMIS *processes* (background, process of recording and analysing data, use of data, monitoring and evaluation of data systems and coordinating mechanism within HMIS) and HMIS *outputs* (dissemination and utilisation of data) [21].

Data collection included secondary document review and informal interviews with five (out of the eight contacted) HMIS staff based at national level which were sampled purposively based on their direct experience of working with mental health (MH) indicators or health information systems in India. The secondary document review was based on government reports, WHO Atlas 2014 data and meeting reports. The government reports mainly included programme specific annual reports or meeting minutes which were available in the public domain. Two Emerald researchers were partly involved in both document review and informal interviews in this process of data collection.

These data were combined and populated in a spreadsheet. Reflecting the domains captured by the situational analysis tool, these data were considered in terms of the three previously stated broad themes of health *inputs, processes and outputs*. Data were furthermore categorised into nine sub-themes which were also based on the details captured by the situational analysis tool and the informal interviews. Wherever there was no or no easily accessible documentation (for example government reports) available, informal interviews were the key source of information. For example, information on the sub themes on use of HMIS data and currently sanctioned and filled HMIS specific posts was collected primarily through informal interviews. These sub-themes were: i) human resources, ii) availability of mental health indicators under the *input theme*; iii) HMIS background, iv) process of recording and analysing data, v) use of HMIS data, vi) monitoring and evaluation of data systems and vii) coordinating mechanism within HMIS within the *process theme* and viii) dissemination; and ix) utilisation of data elaborating the *output* theme.

Indexing and sorting of these data into the spreadsheet was based on these themes and sub-themes. Data coded under each heading were subsequently checked for coherency and completeness by the researchers. From the interviews and the document review researchers derived at a fully populated spreadsheet, with information included regarding each HMIS domain. The process of analysing data involved coding the responses of each question of the situational analysis tool by indexing the data under the themes and subthemes in an excel spreadsheet. Thematic summarisation of the codes resulted in a reduced number of headings which were used to develop summary tables presented in the results section. In order to check whether these headings made sense, independent validation checks were performed by a different senior researcher.

#### Phase 2: Prioritisation exercise

Context is crucial to setting priorities [16], and in this study in India local experts were invited to participate in an expert panel to formulate and prioritise mental health indicators for Sehore district.

The work commenced with an initial phase of establishing a steering committee, constituting four mental health researchers. This expert group was primarily involved in conceptualizing indicators which were appropriate for routine mental health care in India. This list of key indicators was grouped in to the four domains, namely: needs, utilisation, quality and financial protection. This described in more depth in our cross-country paper [17].

These domains include two crucial elements of effective coverage and financial protection. Effective coverage is defined as the number of people in need of services receiving quality care with intended benefits [12]. Effective coverage is mapped by including indicators on need of service, utilisation of services, and quality of care. Financial protection on the other hand is mapped by number of households protected financially while using MH services.

**Fig. 1:**
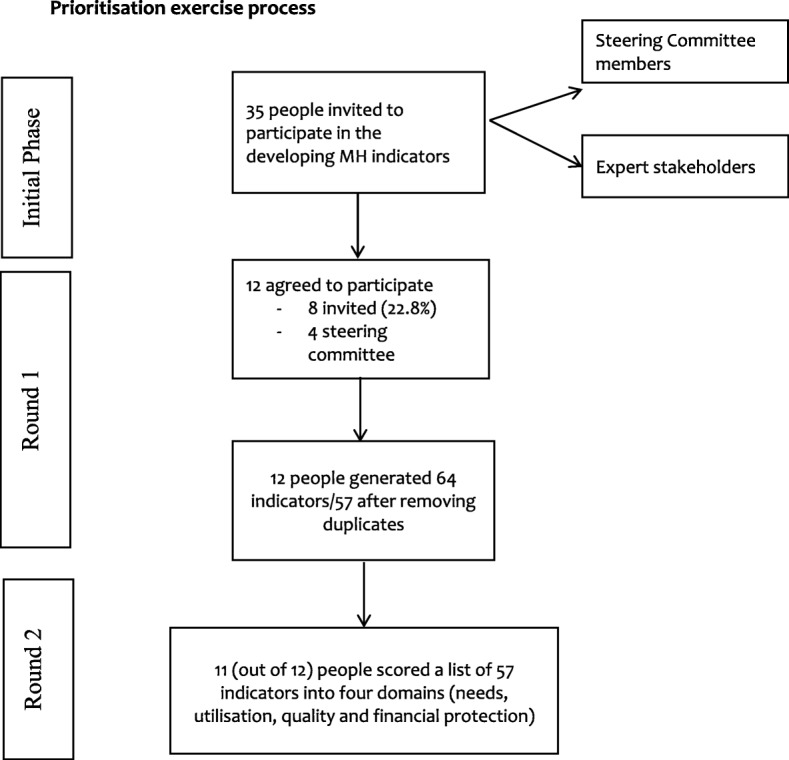
Prioritisation exercise process

In Round 1 of the prioritisation exercise, a group of service providers (district level), public health professionals (state and district level), researchers and service user/caregiver organisations (state and national level) were approached via email to participate in the study. A total number of 35 individuals were invited, out of which n=12 (including n=4 steering committee members) individuals agreed to participate. These participants were all either directly involved in the development/implementation of mental health policy/plans or were advocates for better mental health service delivery. Figure 1 provides an overview of the prioritisation exercise process.

Respondents generated and/or chose potential indicators of mental health service coverage and performance for India.

In Round 2 of the prioritisation exercise, the summarised list (based on four domains) of the potential indicators generated in Round 1 was circulated back to the participants, who were asked to rate these indicators against three equally weighed parameters - significance, relevance and feasibility.

These parameters were rated using a five-point Likert scale (ranging from 1 = strongly disagree to 5 = strongly agree, including a neutral ‘no answer [not informed well to answer]’ option).

Significance was scored by assessing the importance of an indicator; relevance by perceiving if the indicator would influence policy and planning decisions and scores for feasibility were marked by assessing possibility of routinely measuring an indicator. Significance, relevance and feasibility were scored for next 5-10 years by the respondents. A descriptive analysis was carried out in excel and mean rating scores were calculated. All blank responses were excluded both from the numerator and the denominator. After rating the number of essential and minimum indicators (named dashboard indicators) was restricted to 15 as it is difficult to collect more and more data.

#### Phase 3 Consultative workshops

Phase 1 of the study detailed the context of health information system and the situation of mental health routine data. Phase 2 of this study led to a set of indicators on mental health arranged as per expert ratings regarding these indicators’ significance, relevance and feasibility. This was followed by consulting stakeholders on the actual use of these indicators by the stakeholders in Phase 3.

In Phase 3 two consultative workshops regarding these indicators were conducted with the stakeholders at state and national level to determine how to best implement these indicators, mainly focusing on feasibility of indicator implementation. The national-level workshop was conducted in English; however, the state-level workshop was conducted predominantly in the local Hindi language.

Consultative workshops with the stakeholders is a method of qualitative research focusing more on identifying and understanding the context of a problem [22]. Data collection through consultative workshops has been associated with participatory and action research involving theories of enquiry [22].

The Phase 3 stakeholder workshops consisted of 8 (first workshop) and 10 members (second workshop) and involved medical officers (n1*=3, n2*=1) nurses (n1*=1, n2*=0), data management personnel (n1*=1, n2*= 1), psychiatrist (n1*=1, n2*=3), psychologist (n1*=1, n2*=3) and members from Directorate of Health Services (n1=1, n2=2), in the state of Madhya Pradesh. n1* is the number of individuals in 1^st^ workshop and n2* is the number of individuals I the 2^nd^ workshop. These members were purposively selected based on their direct involvement in either planning or implementation of mental health services. In these workshops, the participating stakeholders were first presented with information on the type of indicators generated and the process involved in the earlier stage of this research. The workshop participants were then divided into two groups. These groups were asked to provide insights on how to review, adapt or fine-tune the set of selected indicators that is, the set of 15 indicators developed after prioritisation exercise. Results from each group was then shared with the wider group. Further rethinking on disagreements within the small groups and then wider groups finally produced a consensus regarding which indicators were considered feasible to implement which was the main aim of Phase 3.

The responses from each selected indicator was summarised using a reporting format. This included information on the job description/role description of the respondents, feedback on the feasibility of use and its relation to the existing health management information system and addition/removal of items from the prioritisation exercise. Information was analysed using qualitative techniques. Open descriptive codes were initially applied throughout the data which were further grouped into conceptual codes.

## Results

### Phase 1 Situational Analysis

The results from the situational analysis are summarised below in Table 1. The results are categorized as; governance with a focus on mental health and its data systems, human resources needed for data system management, mental health indicators in HMIS and components within routine HMIS such as data collection, reporting, analysis and dissemination.

**Table 1:** Results from situational analysis checklists

	Health System Components	Response
1	Governance with a focus on mental health and its data systems	
a.	Existence of Mental Health policy and plan	Present
b.	Provision of HMIS in Mental Health Policy	Yes, in the Mental Health Policy draft
c.	General health policies which govern Health Management Information System/HMIS	Yes, in draft National Health policy
d.	General health plans that govern HMIS	Yes, National Rural Health Mission
e.	Standard Operating procedures for Mental Health	No
f.	Initiatives to develop Mental Health Information Systems	No, except for its mention in the draft policy
2	Human Resources	
a.	Minimum qualification to be an HMIS staff	Graduate in any discipline
b.	HMIS expert qualification	BSc/MSc (Bachelors in Science/ Masters in Science) in Statistics
c.	Number of HMIS specialists (at national level)	20
d.	Number of HMIS trainers	Not available
e.	Training manuals for HMIS	Present
f.	Specialised courses in HMIS	No
3	Data Systems (MH indicators in HMIS)	
a.	Mental Health indicators in national HMIS	No^a^
b.	Mental Health Out Patient Department attendance included in HMIS	Yes, at tertiary level in some states
c.	Mental Health referrals recorded	No
d.	Psychiatric inpatient bed occupancy rate	No
e.	Mental health training data reflected	No
f.	Average length of stay at hospital	No
3.a	Components within routine HMIS	
a.	Data collection	Paper and pencil below Primary Health Centre/PHC, electronic in PHC/CHCs and above
b.	Data compilation	HMIS web portal
c.	Data Analysis	Monthly
d.	Frequency of data reporting to Ministry of Health (Tanzania Ministry of Health and Social Welfare)	Monthly, Quarterly and Annually
e.	Data quality control mechanisms	Yes (Supervision, Audits)
f.	Feedback mechanism to the lowest level	Yes, no implementation on ground
g.	Dissemination of HMIS data	Yes, not involving data collection staff
h.	Public access to HMIS report	Yes

^a^However, the revised National Health Information System (2017) has indicators on mental health service delivery at outpatient level

An HMIS operates in India running within the existing health programmes. However, there is no separate policy specific for HMIS, although various health policies such as the new National Health Policy 2017 [23] and new mental health policy 2014 [24] emphasise the importance of an integrated health information systems for routine monitoring. New National Health Policy includes a commitment to integrated information systems by developing linking systems into a common grid.

In terms of the human resources, there exists a staff limitation across levels for managing HMIS. HMIS staff are interdisciplinary and they often manage reporting for various health programmes. Training manual for HMIS exist and are widely used across levels. The general HMIS contains little to no information on mental health. However, some aspects of indicators such as suicide at tertiary hospital level are reported. It was reported that there is no HMIS personnel managing routine reporting for mental health either at the national or state level in India.

### Phase 2 Prioritisation exercise

A total of 35 experts including mental health researchers (n=5), psychiatrists (n=8), psychologists (n=2), programme managers (n=14), and HMIS specialists (n=6) were invited to rate indicators for mental health service delivery and performance.

Round 1 of the prioritisation exercise generated 64 indicators against the four domains of needs, utilisation, quality and financial protection. After removing duplicates, a total number of 57 indicators remained. These were subsequently rated for significance, relevance and feasibility in Round 2. Mean priority score for these indicators ranged from 2.65 to 4.47, with higher values signifying greater agreement on the Likert scale.

This scoring resulted in a list of the most frequently endorsed 15 indicators, covering domains of measuring mental health treatment coverage, including needs, utilisation, quality and financial protection (mean priority scores ranging from 4.48 to 3.78) (see Table 2).

**Table 2:** Results of Prioritisation exercise

Serial No.	Domain	Indicator	Mean Score
1	Utilisation	Number of people with any mental disorder who received mental health treatment by specialist in a given clinic	4.48^b^
2	Need	Number of people diagnosed with severe mental disorders	4.43^b^
3	Need	Number of all people diagnosed with any mental disorder	4.24^b^
4	Quality	Number of days in last one month that psychotropic medicines were out of stock	4.19^b^
5	Quality	Number of persons taking psychotropic drugs	4.14
6	Quality	Number of trained mental health workers at inpatient and outpatient service	4.14^b^
7	Quality	Rate of perceived stigma and discrimination among service users and caregivers	4.05^a^
8	Needs	Rate of suicide deaths and attempts in a given clinic	3.95^b^
9	Utilisation	Number of people with any mental disorder with moderate to severe dysfunction who received mental health treatment in a given clinic	3.95^b^
10	Financial coverage	Number of people with mental disorders who have some kind of financial protection or insurance against the cost of mental health care treatment	3.95
11	Utilisation	Number of people with severe mental disorder who received mental health treatment in a given clinic	3.90^b^
12	Utilisation	Number of people detected by community workers who came to a health care facility for treatment	3.86^a^
13	Utilisation	Number of patients re-admitted to in-patient mental health care	3.81^b^
14	Financial Coverage	Out of pocket expenditures for services as a proportion of household income or spending	3.81
15	Quality	Number of people who score above a validated cut-off score for any mental disorder on self-report checklist (based on national health survey)	3.78^a^

^a^These indicators were different from the cross-country level results. Rest all 12 indicators were similar

^b^List of indicators for inclusion in the mental health information system of Sehore District

Indicators covering both service delivery and health system’s building blocks emerged in the final 15 set of indicators: namely, these included 3 indicators for need, 5 for utilisation, 5 for quality, and 2 for financial protection.

### Phase 3 Consultative workshops

Themes critical for both the content and the context of indicator implementation emerged from the workshop notes. These mainly included: a final indicator list and reflections pertaining to decentralisation of mental health information systems, integrated mental and physical health routine systems, stakeholder involvement, and monitoring and evaluation of these indicators.

Respondents in the consultative workshop at the state level mostly consisted of medical officers who highlighted the need for a local monitoring system for mental health where they can understand the burden of mental health in their catchment area. The need to have additional personnel for data management at each sub-district hospital was brought up by many respondents. Some anticipated that mental health indicators should be integrated at national level in the health management information system to avoid duplication of work at the sub-district hospitals, which are often poorly capacitated in terms of health workforce. The process of involving local experts such as medical officers and health managers before finalising indicators was much appreciated by all the respondents. The role of the State Mental Health Society and the State Mental Health Programme staff were highlighted as crucial in the facilitation of implementation of the proposed indicators.

Experts proposed to reduce the number of indicators assessing quality of care. Whilst our study initially found four quality indicators amongst the 15 highest rated ones (measuring: status of psychotropic medicines in stock, actual number of people taking prescribed drugs, rate of perceived stigma by users, and trained mental health workforce), these were reduced to 2 indicators (measuring: trained staff status, and status of psychotropic drugs in stock) following the discussion during consultative workshops.

As a result of the consultative workshops it was concluded that out of the top 15 dashboard indicators produced via the prioritisation exercise, 9 were foreseen to be feasible and suitable for routine collection in the health care facilities without any immediate additional support. These reflected three indicators on need, two on quality, four on utilisation, to be included for future routine data collection (listed as ** in Table 2).

## Discussion

This study marks one of the first efforts to develop a set of indicators for routine monitoring of mental health services for Sehore district of Madhya Pradesh, India. Using situational analysis, a prioritisation exercise and consultative workshops we identified nine indicators covering domains such as need for treatment, utilisation of care, quality of care and financial risk protection to measure a district’s health system performance for mental health.

These nine indicators were concluded to be immediately implementable at the primary care facilities in Sehore district of Madhya Pradesh, without any additional support. However, a functional district mental health programme and other research and implementation projects including Emerald arranged for a platform for delivering MH services at these primary care facilities. Implementation of these indicators will include training of health workers, managers and doctors involved in the mental health service delivery, procurement of registers for record keeping, and developing guides for indicator implementation (e.g. through adding a time bound component to indicators such as re-admission rates).

Notably, the routine information system for mental health in India is weak and national mental health surveys have been the major source of mortality and morbidity data [25].

The need for strengthening routine data collection is even more pressing now as countries are moving towards an independent self-sustaining health model from an international agencies led/supported model.

The Indian government’s modest initiative to strengthen mental health services at primary care level through a district mental health programme has faced various implementation challenges. A lack of clinical skills to diagnose and treat mental disorders in a resource-poor? environment with limited mechanisms to track, refer and follow up patients have been documented. [11]. This is coupled by marginal reporting of indicators to track performance which is predominantly capturing data from tertiary hospitals [4].

Our study has filled these gaps in monitoring by producing context specific indicators for the primary care level where mental health services are also delivered. These feasible indicators cover both health service delivery aspects with indicators on coverage and health system aspects measuring length of stay, medicines in stock and bed occupancy rates. The latter are fundamental to monitoring services but are often missing.

Even though crucial to be reported, indicators on suicide rates and attempts, daily stock-out rates of medicines and rehospitalisation need a robust system in place which in this case is provided by a functional district mental health programme and research projects. Therefore, an ongoing implementation and evaluation of these indicators is needed to ensure sustainability of these indicators.

Substantial advancements in meeting information needs for mental health over the last decade have been reported in the literature. On one hand estimates from disease burden [26] pushed for a need to report on mental health conditions in the countries, on the other hand initiatives by international organisations including the WHO Assessment Instrument for Mental Health Systems [27], Mental Health Action Plan 2030 [7], WHO Atlas 2017 [5] and quality of mental health care indicators by the Organisation of Economic Co-Operation and Development [28] made provisions to make reporting easier.

Overloaded HMIS in countries demand contextualised and feasible mental health indicators to be included in routine reporting. Similar to our study, realistic measures to develop and strengthen mental health indicators were reported in Nigeria [8] and other LMICs [14]. It has been argued that the absence of quality mental health care indicators is one of the reasons behind poor evidence on mental health performance [29]. This study used a feasible and sustainable approach in developing indicators measuring mental health service delivery at facility-level. Similar measures can be adapted to include quality indicators in routine monitoring in comparable settings.

Measures to reduce the mental health treatment gap in LMICs involves scaling up mental health services [30]. Even though such community scale up measures have been underway in India since the 1980s, strengthening information systems can augment the process of measuring effective coverage by estimating the outcome data for those treated [12].

### Limitations

The results of this study need to be interpreted in view of a number of limitations. While our approach of developing indicators may be valid across similar settings, the exact indicators recommended may not be generalisable to all other states of India and other LMICs. This study is also limited by its ability to draw conclusions form the situational analysis checklist, due to the scarcity of resources available in the literature based on which these conclusions were formulated. However, the use of interviews coupled with document review enabled us to respond to such gaps in the literature. The response rate in the priority setting exercise was low (22.8%), although the respondent retention rate from Round 1 to Round 2 was high (91%). Difficulty in getting experts to participate in the study might be due to the less familiar or less trustful electronic way of reaching out to the experts, a lack of interest/knowledge in the area, or the lack of time. However, an electronic way of contacting experts meant that more people could be contacted across a large geographical area, and this approach also provided experts flexibility in view of their busy schedules.

Consultative workshops as used in Phase 3 of this study limit the ability to remain impartial, due to the potential social desirability bias where some views may dominate over others (especially if driven by seniors in the team). Also, our focus has been development of mental health indicators for primary care facilities within the public sector. This study does not explore mental health indicator needs of private providers as this was beyond the scope of this work. Again, further research is needed to assess the implementation of these indicators over time to validate their sustainability in the public mental health systems. Evaluation of the implementation of these indicators is underway and will be reported in an upcoming publication.

## Conclusion

This study drew on situation analysis, consensus building, consultative processes and elements of action research, in which local experts take part in the prioritisation and planning in the development of indicators for routine monitoring of mental health services in primary health care. We generated, prioritised and selected nine mental health indicators that can be used to examine whether people with mental illnesses are effectively covered by the public mental health services. Mental health data is never prioritised in HMIS within LMICs, yet mental disorders are now recognised to contribute to a considerable burden of disease and disability and are an important public health concern. With many countries undergoing strengthening of their mental health? information systems to meet the national and international information needs, countries can utilise the phased approach developed in this study to develop context-specific key indicators measuring both mental health service delivery and system performance.

## Abbreviations

HMIS: Health Management Information Systems
ICD- 10: 10^th^ International Classification of Diseases
LMIC: Low and Middle-Income Countries
MH: Mental health
PHC: Primary Health Centres
WHO: World Health organisation
